# A complex water network contributes to high-affinity binding in an antibody–antigen interface

**DOI:** 10.1016/j.dib.2015.12.023

**Published:** 2015-12-19

**Authors:** S.F. Marino, D. Olal, O. Daumke

**Affiliations:** Max Delbrueck Centrum for Molecular Medicine, Berlin, Germany

**Keywords:** Crystal structure, Fab fragment, BCMA, Binding interface, High affinity, Water molecules

## Abstract

This data article presents an analysis of structural water molecules in the high affinity interaction between a potent tumor growth inhibiting antibody (fragment), J22.9-xi, and the tumor marker antigen CD269 (B cell maturation antigen, BCMA). The 1.89 Å X-ray crystal structure shows exquisite details of the binding interface between the two molecules, which comprises relatively few, mostly hydrophobic, direct contacts but many indirect interactions over solvent waters. These are partly or wholly buried in, and therefore part of, the interface. A partial description of the structure is included in an article on the tumor inhibiting effects of the antibody: “Potent anti-tumor response by targeting B cell maturation antigen (BCMA) in a mouse model of multiple myeloma”, Mol. Oncol. 9 (7) (2015) pp. 1348–58.

Specifications Table

**Table:** 

Subject area	*Chemistry, Biology, Cancer immunology*
More specific subject area	*Structural biology*
Type of data	*Parameter table for structurally observed water molecules; figures depicting positions of these waters in a binding interface*
How data was acquired	*Analysis of the refined X-ray crystallographic structure.*
Data format	*Table, figures.*
Experimental factors	*Fab fragments from anti-CD269 (BCMA) IgGs co-crystallized with antigen and the structure solved to high (1.89 Å) resolution*
Experimental features	*Interface interactions in the refined structure were assessed and the contribution of water to the binding evaluated.*
Data source location	*Protein Data Bank*
Data accessibility	〈http://www.rcsb.org/pdb/home/home.do〉 (*PDB code:* 4*ZFO*)

## Value of the data

• The antibody targets the very restrictively expressed tumor marker BCMA for Multiple Myeloma and potently inhibits tumor growth in mice
• The antibody has an exceptionally high affinity to BCMA (picomolar)
• The high resolution crystal structure of the antibody-BCMA complex reveals a network of water molecules in the binding interface
• The structure shows how very high affinity can be achieved with a minimal number of direct protein-protein contacts

## 1. Data, experimental design, materials and methods

Multiple Myeloma (MM) is an incurable malignancy of antibody-secreting B cells (plasma cells) with a mean life expectancy of 5 years from diagnosis [1]. We generated a chimeric mouse/human antibody (J22.9-xi) against CD269 (BCMA), a plasma membrane receptor expressed nearly exclusively in plasma cells; the antibody shows potent cell killing activity on MM cells *in vitro* and anti-tumor activity *in vivo*. Surface Plasmon Resonance Spectrometry (SPR) measurements gave an exceptionally high affinity of J22.9-xi for BCMA of 50 pM [2]. Although higher affinity antibody:antigen interactions have been achieved by protein engineering/selection techniques [3], the J22.9-xi interaction exceeds the low nanomolar affinity range typical of therapeutic antibodies [4], [5] (see also, for example, Herceptin at 5 nM [6] and Rituximab at 8 nM [7]) by approximately 100-fold.

**Fig. 1:**
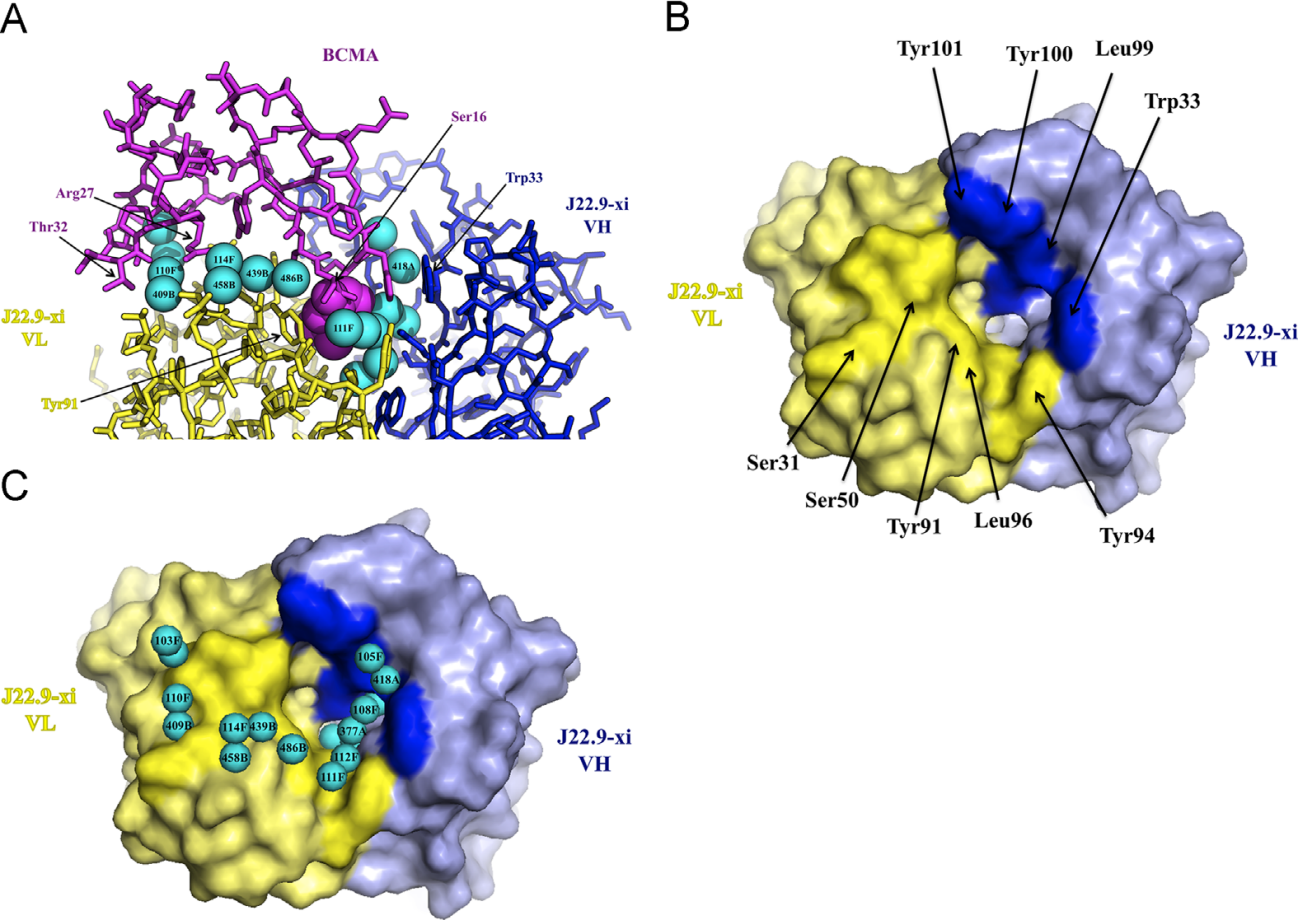
Water molecules in the J22.9-xi binding site. **A.** A view of the binding interaction between BCMA (magenta) and the variable domains of the heavy chain (VH, blue) and light chain (VL, yellow). Interface water molecules participating in bridging hydrogen bonding interactions between BCMA and J22.9-xi are depicted as cyan spheres with a radius of 1.4 Å. For clarity only some interface residues (with the corresponding chain color) and some interface waters (with chain numbers directly on the spheres) are labeled. Leu17 in the BCMA *Dx*L loop is shown in space-filling representation. **B.** A view looking down on the binding cavity in J22.9-xi with BCMA removed. The Fab fragment is shown in surface representation with the heavy chain colored blue and the light chain colored yellow. Some of the residues making direct contacts to BCMA are indicated with arrows and darker coloring. **C.** View as in (**B**) with interface waters depicted as cyan spheres and labeled as in (**A**).

A protruding loop in the antigen BCMA (the D*x*L loop) with Leu17 at its apex is critical for binding to the native BCMA ligands, B cell Activating Factor of the TNF Family (BAFF) and A PRoliferation Inducing Ligand (APRIL) [8], both of which have a hydrophobic cavity that packs tightly around Leu17. Our 1.89 Å X-ray structure of a J22.9-xi Fab fragment in complex with BCMA shows that J22.9-xi also targets the D*x*L loop (Fig. 1A). However, the Leu17 binding cavity in J22.9-xi is substantially larger than the Leu17 side chain and provides only limited hydrophobic interactions between Leu17 and the VL of the antibody, primarily with Tyr91; the remainder of the binding cavity is filled with 6 water molecules that pack against the side of Leu17 opposite the Tyr91 interaction (Fig. 1A, B and C). This high affinity interaction comprises few minor direct contacts between the molecules (4 direct hydrogen bonds and 21 mostly single atom van der Waals contacts) but at least 50 hydrogen bonds involving waters “bridging” interactions between main chain and side chain atoms; 17 of these water molecules are directly in the binding interface (Table 1). Thus, the structure shows how high affinity binding can be achieved with only a small number of direct interactions between the binding partners.

## 2. Analysis of binding interface

Direct atom to atom contacts between BCMA and the VH and VL of J22.9-xi were identified using the PISA interaction server [9]. Direct and indirect hydrogen bonding interactions involving water were identified and validated (distance and geometry) from the structure using LigPlot [10] and COOT [11].

## Figures and Tables

**Table 1:** Water interactions in the J22.9-xi:CD269 complex. The interaction partners are read from left to right across the table, with the corresponding H-bond distances and thermal displacement (B) factors of the indicated water molecules listed (J22.9-xi:H_2_O:CD269). For example, Ser31 from the J22.9-xi light chain participates in a hydrogen bonding interaction with water 409B at a distance of 2.50 Å and water 409B in turn interacts with Thr32 of CD269 at a distance of 2.65 Å. The data in Table 1 were generated using the software LigPlot [8] and COOT [11].

**Light Chain (B)**	**H**_**2**_**O#**	**CD269 (F)**	**Distance (Å)**	**B (Å**^**2**^**)**
Ser31 (sc)	409B	Thr32 (sc, mc)	2.50, 2.65 (sc)	24.33
2.50, 3.15 (mc)
	409B, 110F	Arg27 (sc)	2.65, 2.60, 2.98	24.33, 23.47
	409B, 110F	Ser30 (sc)	2.50, 2.65, 2.69	24.33, 23.47
Ser31 (mc)	458B, 114F	Arg27 (sc)	2.71, 2.75, 2.78	26.48, 28.32
Asn32 (sc)	439B	Asp15 (sc)	2.67, 2.88	19.24
	439B, 114F	Arg27 (sc)	2.67, 2.94, 2.78	19.24, 28.32
	486B	Ser16 (sc)	2.80, 3.18	15.45
Tyr36 (sc)	522B, 336A, 377A	Leu17 (mc)	3.19, 2.90, 2.77, 2.88	14.87, 18.33, 35.33
Ser50 (sc)	439B	Asp15 (sc)	3.11, 2.88	19.24
Ser52 (sc)	110F	Ser30 (sc)	2.73, 2.69	23.47
	110F	Arg27 (sc)	2.73, 2.98	23.47
Ser52 (sc)	479B, 103F	Ser30 (mc)	2.78, 2.65, 2.94	25.34, 31.49
Ser29 (sc)	2.78, 2.65, 2.54
	110F, 409B	Thr32 (sc, mc)	2.73, 2.60, 2.65	23.47, 24.33
Gly66 (mc)	409B	Thr32 (sc)	2.86, 2.65	24.33
	409B, 110F	Arg27 (sc)	2.86, 2.60, 2.98	24.33, 23.47
	409B, 110F	Ser30 (sc)	2.86, 2.60, 2.69	24.33, 23.47
Gln89 (sc)	522B, 336A, 377A	Leu17 (mc)	2.92, 2.90, 2.77, 2.88	18.47, 18.33, 35.33
Tyr91 (mc)	111F	Ser16 (sc)	2.89, 2.71	32.32
	111F, 112F	Ser16 (mc)	2.89, 2.79, 2.76	32.32, 23.28
Tyr94 (sc)	112F	Ser16 (mc)	3.12, 2.76	23.28
	112F, 111F	Ser16 (sc)	3.12, 2.79, 2.71	23.28, 32.32
				
**Heavy Chain (A)**	**H**_**2**_**O#**	**CD269 (F)**	**Distance (Å)**	**B (Å2)**
Trp33 (mc)	421A, 108F	Leu17 (mc)	2.98, 2.76, 2.62	16.25, 31.34
	418A	Leu18 (mc)	2.90, 3.45	19.66
Ser35 (sc)	421A, 108F	Leu17 (mc)	2.91, 2.76, 2.62	16.25, 31.34
336A, 377A
2.64, 2.77, 2.88	18.33, 35.33
Trp47 (sc)	336A, 377A	Leu17 (mc)	2.96, 2.77, 2.88	18.33, 35.33
Glu50 (sc)	112F	Ser16 (mc)	3.21, 2.76	23.28
	112F, 111F	Ser16 (sc)	3.21, 2.79, 2.71	23.28, 32.32
	377A	Leu17 (mc)	2.75, 2.88	35.33
	377A, 108F	Leu17 (mc)	2.75, 2.48, 2.62	35.33, 31.34
Leu99 (mc)	108F	Leu17 (mc)	3.26, 2.62	31.34
Tyr101 (mc)	105F	Leu18 (mc)	3.50, 2.57	25.20
